# Inhibiting aberrant p53-PUMA feedback loop activation attenuates ischaemia reperfusion-induced neuroapoptosis and neuroinflammation in rats by downregulating caspase 3 and the NF-κB cytokine pathway

**DOI:** 10.1186/s12974-018-1271-9

**Published:** 2018-09-01

**Authors:** Xiao-Qian Li, Qian Yu, Feng-Shou Chen, Wen-Fei Tan, Zai-Li Zhang, Hong Ma

**Affiliations:** 1 Department of Anesthesiology, First Affiliated Hospital, China Medical University, Shenyang, 110001 Liaoning China; 2 Department of Thoracic Surgery, Fourth Affiliated Hospital, China Medical University, Shenyang, 110032 Liaoning China

**Keywords:** Apoptosis, Blood-spinal cord barrier, Inflammation, Ischaemia reperfusion, p53, p53 upregulated modulator of apoptosis

## Abstract

**Background:**

Ischaemia reperfusion (IR) induces multiple pathophysiological changes. In addition to its classical role in regulating tumourigenesis, the feedback loop formed by p53 and its driven target p53-upregulated modulator of apoptosis (PUMA) was recently demonstrated to be the common node tightly controlling various cellular responses during myocardial IR. However, the roles of the p53-PUMA feedback loop in the spinal cord remain unclear. This study aimed to elucidate the roles of p53-PUMA feedback interactions in the spinal cord after IR, specifically investigating their regulation of caspase 3-mediated apoptosis and nuclear factor (NF)-κB-mediated cytokine release.

**Methods:**

SD rats subjected to 12 min of aortic arch occlusion served as IR models. Neurological assessment as well as p53 and PUMA mRNA and protein expression analyses were performed at 12-h intervals during a 48-h reperfusion period. The cellular distributions of p53 and PUMA were determined via double immunofluorescence staining. The effects of the p53-PUMA feedback loop on modulating hind-limb function; the number of TUNEL-positive cells; and protein levels of caspase 3, NF-κB and cytokines interleukin (IL)-1β and tumour necrosis factor (TNF)-α, were evaluated by intrathecal treatment with PUMA-specific or scramble siRNA and pifithrin (PFT)-α. Blood-spinal cord barrier (BSCB) breakdown was examined by Evans blue (EB) extravasation and water content analyses.

**Results:**

IR induced significant behavioural deficits as demonstrated by deceased Tarlov scores, which displayed trends opposite those of PUMA and p53 protein and mRNA expression. Upregulated PUMA and p53 fluorescent labels were widely distributed in neurons, astrocytes and microglia. Injecting si-PUMA and PFT-α exerted significant anti-apoptosis effects as shown by the reduced number of TUNEL-positive cells, nuclear abnormalities and cleaved caspase 3 levels at 48 h post-IR. Additionally, p53 colocalized with NF-κB within the cell. Similarly, injecting si-PUMA and PFT-α exerted anti-inflammatory effects as shown by the decreased NF-κB translocation and release of IL-1β and TNF-α. Additionally, injecting si-PUMA and PFT-α preserved the BSCB integrity as determined by decreased EB extravasation and spinal water content. However, injecting si-Con did not induce any of the abovementioned effects.

**Conclusions:**

Inhibition of aberrant p53-PUMA feedback loop activation by intrathecal treatment with si-PUMA and PFT-α prevented IR-induced neuroapoptosis, inflammatory responses and BSCB breakdown by inactivating caspase 3-mediated apoptosis and NF-κB-mediated cytokine release.

## Background

Spinal cord ischemia reperfusion (IR) injury is a major challenge and severe complication during cardiothoracic and vascular surgery [1], as it definitively induces excessive and extensive secondary injuries due to a wide range of pathological changes. Spinal cord IR injury elicits a concomitant increase in blood-spinal cord barrier (BSCB) permeability, accompanied by the subsequent activation of proinflammatory responses and cellular programmed or autophagy-associated death [2, 3]. Commonly, these responses are regulated by separate signalling pathways, and recent emerging evidence indicates that they may coordinate and integrate into a signalling network in response to ischaemia [4, 5]. As a stress sensor under stimuli, the tumour suppressor transcription factor p53 drives the expression of multiple targets to execute p53-mediated cellular functions [6–8]. In addition to its classical role in regulating tumour metastasis, p53 was newly demonstrated to be a common node in a signalling network that initiates apoptosis and inflammation during brain ischaemia [9, 10]. Upon ischaemia, p53 rapidly accumulates in the injured zone and tightly controls the balance between pro-apoptotic proteins (e.g., BH3-interacting domain death agonist, Bid) and anti-apoptotic proteins (e.g., B-cell lymphoma 2, Bcl-2) by promoting a feedback interaction with the murine double minute 2 (MDM2) gene [7, 11]. Moreover, p53 is thought to regulate not only the p53-mediated pathway but also other pathways that participate in various diseases models [6, 12, 13]. Pharmacologically blocking p53 function by pifithrin (PFT)-α induced significant neuroprotective effects by inhibiting nuclear factor (NF)-κB transcription and releasing the downstream proinflammatory cytokines interleukin (IL)-1β and tumour necrosis factor (TNF)-α [14]. In addition to MDM2, p53 upregulated modulator of apoptosis (PUMA) has gradually received substantial attention among a list of p53-driven targets [8, 13, 15]. A BH3-only protein (BH3) in the Bcl-2 protein family, PUMA is expressed at a very low level under normal conditions. PUMA can be induced immediately by upregulation of the p53 level and acts as a crucial checkpoint that finely controls further p53-dependent network activation in different models of diseases [8, 15, 16]. Therefore, regulating the levels and function of PUMA is expected to become a new therapeutic target against IR [13]. In agreement with this hypothesis, silencing PUMA expression using knockout mice or the targeting RNA interference technique significantly decreased its ability to interact with p53 during ischaemic injury, which further prevented against p53 transactivation and cellular apoptosis [15, 17]. Thus, better understanding on how to properly regulate the p53-PUMA feedback loop in vivo may significantly impact the development of new therapies. However, little is known about a common node of p53 and how the p53-PUMA feedback loop functions via both apoptotic and proinflammatory mechanisms during spinal cord IR. In this context, we first elucidated the IR-induced temporal profiles of p53 and PUMA expressed in the spinal cord to investigate the potential interactions in an adult rat model of IR. We then assessed the effects of the p53-PUMA feedback loop on hind-limb motor function; the number of TUNEL-positive cells; and the protein levels of caspase 3, NF-κB, cytokines IL-1β, and TNF-α, in vivo by the intrathecal injection of specific PUMA siRNA (si-PUMA), scramble siRNA (si-Con) and PFT-α, a p53-specific inhibitor. Additionally, the effects of si-PUMA and PFT-α on preventing BSCB leakage were evaluated to further determine the inflammatory responses after IR.

## Methods

### Animals

Eight-week-old Sprague Dawley (SD) rats, weighing approximately 250 g, were purchased from the Animal Center of China Medical University (Shenyang, China). The rats had free access to food and water and housed in a standard cage that was maintained at 22–24 °C, relative humidity of 50–60% with a 12/12-h light/dark cycle for 1 week before surgery.

### Rat model of spinal cord IR injury

The rat IR model was established as we previously reported [18]. After anaesthetization via an intraperitoneal injection of 10% chloral hydrate (300 mg/kg body weight; Beyotime Biotechnology, Shanghai, China), the rats were mechanically ventilated by endotracheal intubation. Two 24-gauge catheters were separately inserted into the left carotid artery or the tail artery to measure the proximal and distal blood pressure, respectively. By directly envisioning the left thoracotomy, the aortic arch was exposed and clamped just distal to the left subclavian artery to induce ischaemia, which was confirmed by the observation of a distal blood pressure less than 10 mmHg. After 12 min, the clamp was removed to initiate the 48 h reperfusion period. Rats in the sham group were subjected to the same surgery without clamping.

### Intrathecal injection of PUMA siRNA and control

The rat sequences of si-PUMA and si-Con were constructed by Genechem (Shanghai, China) according to effective interference sites previously reported [16]. The sequences of si-PUMA and si-Con are as follows: si-PUMA, 5′-CGUGUGACCACUGGCAUUC-3′ and 5′-GAAUGCCAGUGGUCACACG-3; and si-Con, 5′-UUCUCCGAACGUGUCACGU-3′ and 5′-ACGUGACACGUUCGGAGAA-3′. si-PUMA and si-Con were transfected in vivo via intrathecal injection together with Lipofectamine 3000 (Invitrogen, CA, USA) as reported previously [18]. The injection position was located at the L_5–6_ segments of the dura. Each injection was administered at the same time of day, and the correct position was confirmed by the tail flick phenomenon [18]. After three consecutive injections, PUMA mRNA expression levels were detected to determine the optimal interference concentration from four adjacent concentration gradients (50, 100, 200 and 300 μmol/L). Based on the above, 100 μmol/L was selected as the final concentration because it was the lowest concentration that significantly downregulated PUMA mRNA expression. In addition, only rats with normal motor function after three injections were included in this study.

### Experimental protocol

**Fig. 1:**
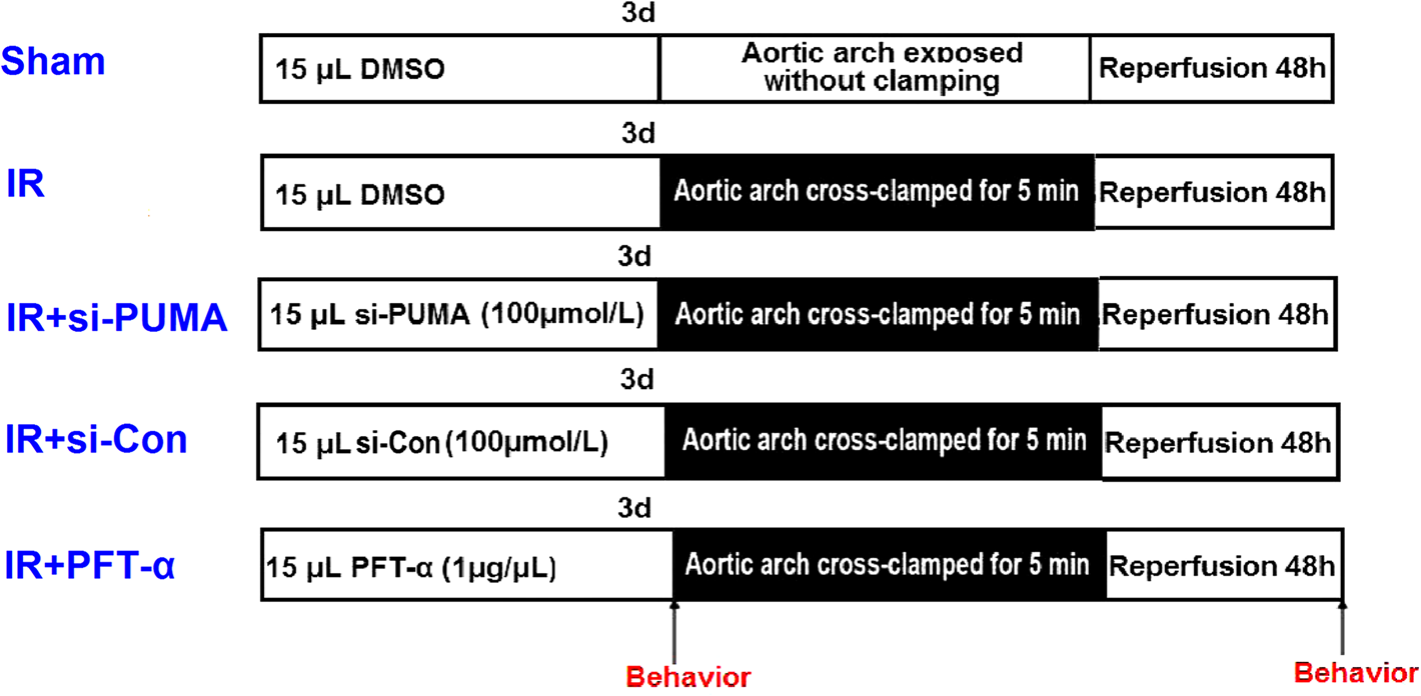
Experimental groups and protocol. Schematic diagram of the five groups of rats exposed to different experimental treatments

The rats were randomly assigned into groups (*n* = 6 per group) as shown in Fig. 1. The sham group rats were subjected to the surgical procedure without ischaemia, and rats in all other groups underwent ischaemia after three consecutive intrathecal injections. Each injection contained 15 μL of total fluid comprising 2% dimethyl sulfoxide (DMSO), si-PUMA, si-Con, or PFT-α (a p53 inhibitor, 1 μg/μL, Selleck Chemicals, S2929, PA, USA). Rats were euthanized at 48 h after surgery with an overdose of sevoflurane. Spinal cord segments L_4–6_ were collected for further analysis.

### Neurological assessment

For 48 h post-IR, hind-limb neurological functions were detected at 12-h intervals using modified Tarlov criteria. The functions were quantified by ambulation and ranged from 0 (no ankle movement) to 4 (normal). The assessments were performed by two observers who were blinded to the entire experimental protocol [19].

### Quantification of p53 and PUMA mRNA expression

Total RNAs from dissected L_4–6_ spinal cord segments were extracted using TRIzol reagent (Beyotime, Beijing, China). First-strand cDNAs were transcribed using the PrimeScript RT Reagent Kit (Takara, Tokyo, Japan), incubated with the corresponding primers and SYBR Green SuperMix-UDG (Thermo Fisher Scientific, MA, USA), and analysed on a Prism 7000 detection system (Applied Biosystems, CA, USA). Each gene was quantified in triplicate, and the levels were normalized to those of β-actin. Data were analysed using the 2^-ΔΔCt^ method. The following primers were used: p53 (forward, 5′-CCCAGGGAGTGCAAAGAGAG-3′ and reverse, 5′-TCTCGGAACATCTCGAAGCG-3′); PUMA (forward, 5′-GTGTGGAGGAGGAGGAGTGG-3′ and reverse, 5′-TCGGTGTCGATGTTGCTCTT-3′); and β-actin (forward, 5′-CAGGAGATGGCCACTGCCGCA-3′, and reverse, 5′-TCCTTCTGCATCCTGTCAGCAC-3′).

### Double immunofluorescence staining

The cellular distribution of PUMA and p53 expression after IR was determined by double immunofluorescence staining at 48 h post-IR, as the lowest behavioural scores were also measured at that time point [18]. Briefly, the spinal cord tissues were frozen and sliced into 20-μm-thick sections. After permeabilization and blocking with 10% bovine serum albumin (BSA), the slices were incubated overnight at 4 °C with the rabbit anti-PUMA (Abcam, ab9643, 1:100, MA, USA) or rabbit anti-p53 primary antibody (Abcam, ab131442, 1:200) and specific cellular marker primary antibodies as follows: mouse anti-NeuN (neuronal marker, Abcam, ab104224, 1:500), mouse anti-GFAP (astrocytic marker, Abcam, ab10062,1:500) and mouse anti-Iba-1 (microglial marker, Abcam, ab15690,1:400). The samples were then incubated with the secondary reagents Alexa 488-conjugated donkey anti-mouse IgG (1:500, Life Technologies, CA, USA) and Alexa 594-conjugated donkey anti-rabbit IgG (1:500, Life Technologies) for 2 h at room temperature to visualize the staining. Nonspecific staining was excluded by incubation with nonimmune serum but no primary antibody. Representative images of anteriorl horns were captured at 400× magnification on the Leica TCS SP2 fluorescence microscope (Leica Microsystems, Buffalo Grove, IL, USA). Quantified fluorescence intensities and numbers of immunoreactive cells were averaged for three stained slices per antibody.

Additionally, potential interactions among p53, PUMA and NF-κB p65 were also detected exactly as described above with the following primary antibodies: mouse anti-p53 (Abcam, ab26, 1:200), rabbit anti-PUMA (Abcam, ab9643, 1:100), rabbit anti-NF-κB (Abcam, ab16502, 1:100) and mouse anti-NF-κB (Cell Signalling Technology, no. 6956, MA, USA).

### Western blotting

L_4–6_ spinal cord segments were rapidly collected and homogenized on ice due to their vulnerability to ischaemic injury [20]. The cytosolic and nuclear fractions were extracted and purified with a nuclear and cytosol protein extraction kit (KangChen, KC415, Shanghai, China) [18]. The mitochondrial fractionation was performed according to a multiple centrifugation method described previously [15]. Briefly, the homogenates were first centrifuged at 750 g at 4 °C and then at 8000 g for 20 min at 4 °C. The pellets obtained were considered the mitochondrial fraction. The samples were separated by gel electrophoresis and then transferred to polyvinylidene difluoride membranes. After blocking with 5% non-fat milk, the membranes were incubated overnight at 4 °C with primary antibodies against p53 (Abcam, ab26, 1:200), PUMA (Abcam, ab9643, 1:200), cleaved caspase 3 (Cell Signaling Technology, #9661, 1:300) and NF-κB p65 (phospho S536, Abcam, ab86299, 1:100). β-actin (Abcam, ab8227, 1:10000), histone (Abcam, ab10799, 1:5000) and COX-IV (Abcam, ab33985, 1:5000) were used as the controls. The bands were visualized by an enhanced chemiluminescence (ECL) kit (Beyotime, Beijing, China) and quantified using Quantity One software (Bio-Rad Laboratories, Milan, Italy).

### Terminal deoxynucleotidyl transferase-mediated dUTP nick-end labelling (TUNEL) assay

The TUNEL assay was performed to identify cellular apoptosis as previously described [3]. After fixation in 4% formaldehyde, the spinal sections were stained with an ApopTag® Fluorescein In Situ Apoptosis Detection Kit (EMD Millipore, S7110) according to the manufacturer’s instructions. Briefly, the sections were incubated with TUNEL reaction mixture and kept in the dark for 60 min at room temperature. Then, 4′,6-diamidino-2-phenylindole (DAPI, Beyotime) was added for 5 min to label the cell nuclei. Nonspecific staining was determined by omitting the TdT enzyme from the reaction mixture. The average numbers of TUNEL-positive cell in the anterior regions of three spinal sections were counted for comparison among the groups. Samples from each step were washed three times in phosphate buffered saline (PBS).

### Morphological analysis of apoptotic nuclei

The morphology of apoptotic nuclei was visualized by DAPI staining [15]. Briefly, after fixation in 4% paraformaldehyde, the sections were stained with DAPI solution for 2 min at room temperature. Representative images of the anterior horns were captured at 400× magnification, and the number of nuclear abnormalities was averaged in three randomly selected slices.

### BSCB permeability assessment

Evans blue (EB) extravasation and spinal water content analyses were performed to detect the integrity and permeability of BSCB as previously described [18]. Briefly, 2% EB (35 mg/kg, Sigma, MO, USA) was slowly administered via the tail vein 30 min before euthanization. After EB circulated for 20–30 min, the rats were transcardially perfused with 180 mL of sterile saline at 60 mL/min. To detect fluorescence, the L_4–6_ segments were cut into 15-μm sections and visualized under a BX-60 fluorescence microscope (Olympus, NY, USA) with a green filter. For quantitation, the tissues were incubated in 400 μL of N,N′-dimethylformamide at 50 °C for 72 h. After centrifugation, the supernatants were collected and colorimetrically measured at 632 nm. Concentrations were reported as the amount of EB per spinal cord wet tissue weight (microgramme per gram) according to a standard curve.

### Spinal cord Oedema assessment

The water content was measured by wet-dry method to quantify the spinal cord oedema. Fresh tissues from non-perfused rats were immediately collected. Approximately 1 cm of the L_4–6_ segments of the spinal cords was weighed as wet weight. Then, the segments were dried at 110 °C for 24 h and reweighed as dry weight (Havppvel method). The percentage of water content was calculated using the following formula: spinal cord water content (%) = (wet weight − dry weight)/wet weight × 100% [21].

### Measurement of IL-1β and TNF-α by ELISA

The spinal cords were collected, homogenized and centrifuged, and their IL-1β and TNF-α contents were measured with ELISA kits (R&D Systems, MN, USA) according to the manufacturer’s instructions [18]. Absorbance was detected at 450 nm, and the content of each sample was based on the standard curve and expressed as picogram per milligram protein.

### Statistical analysis

All statistical data were normally distributed and presented as the mean ± SEM in this study. When compared between groups, the data were analysed with the *t* test, one-way or two-way ANOVA followed by post hoc Tukey test by SPSS software (version 17.0, SPSS Inc., Chicago, USA). A *P* < 0.05 was considered statistically significant.

## Results

### Temporal induction of p53, PUMA levels and behavioural deficits post-IR

**Fig. 2:**
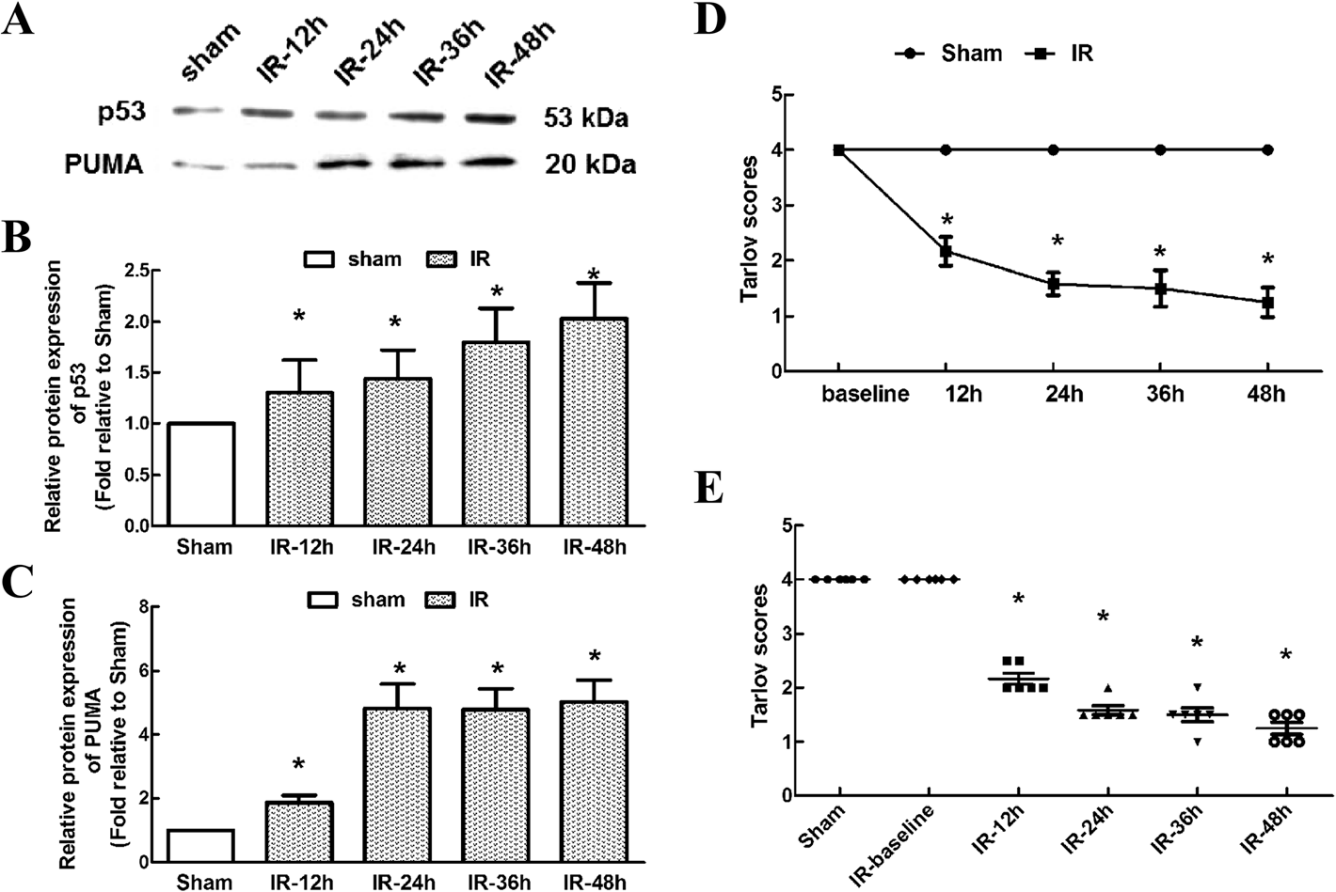
Temporal profiles of IR-induced alteration in p53 and PUMA protein levels and neurological assessments. **a** Representative western blots of p53 and PUMA levels during the 48-h reperfusion period. **b** and **c** Quantification of the integrated intensities of p53 and PUMA bands. Data are expressed as the mean ± SEM. The relative protein expression was normalised to that of the sham group. *P < 0.05, versus the sham group. **d** Neurological assessments were scored using modified Tarlov criteria at 12-h intervals during 48 h of reperfusion. **e** Individual assessments of each rat at the indicated time points. Each symbol represents one mouse. The bar represents the median. Data are expressed as the mean ± SEM. *n* = 6 in each group. *P < 0.05, versus the sham group

Given that p53 and PUMA are required for IR-induced cardiomyocyte apoptosis in vitro [16], we hypothesized that they might partially function in spinal cords in vivo. We detected temporal changes in p53 and PUMA protein expression at 12-h intervals for 48 h post-IR. Compared with those in the sham group, the levels of p53 and PUMA in the reperfusion group significantly increased with time during the 48 h reperfusion period and peaked at 48 h (Fig. 2a–c, *P* < 0.05), suggesting that potential feedback interactions occur during IR.

As expected, during the 48-h reperfusion period, temporal assessments of motor function according to Tarlov scores showed tendencies opposite those shown by the p53 and PUMA levels at all the observed time points. The average Tarlov scores in the IR group were much lower than those in the baseline and sham groups, with the lowest levels being observed at 48 h (Fig. 2d, *P* < 0.05). Similarly, the individual scores for each rat shown in Fig. 2e confirmed the development of neurological dysfunctions after IR (*P* < 0.05). In addition, this coincidence indicated the involvement of p53-PUMA in IR-induced behavioural deficits.

### Cellular distribution of IR-induced p53 and PUMA expression post-IR

**Fig. 3:**
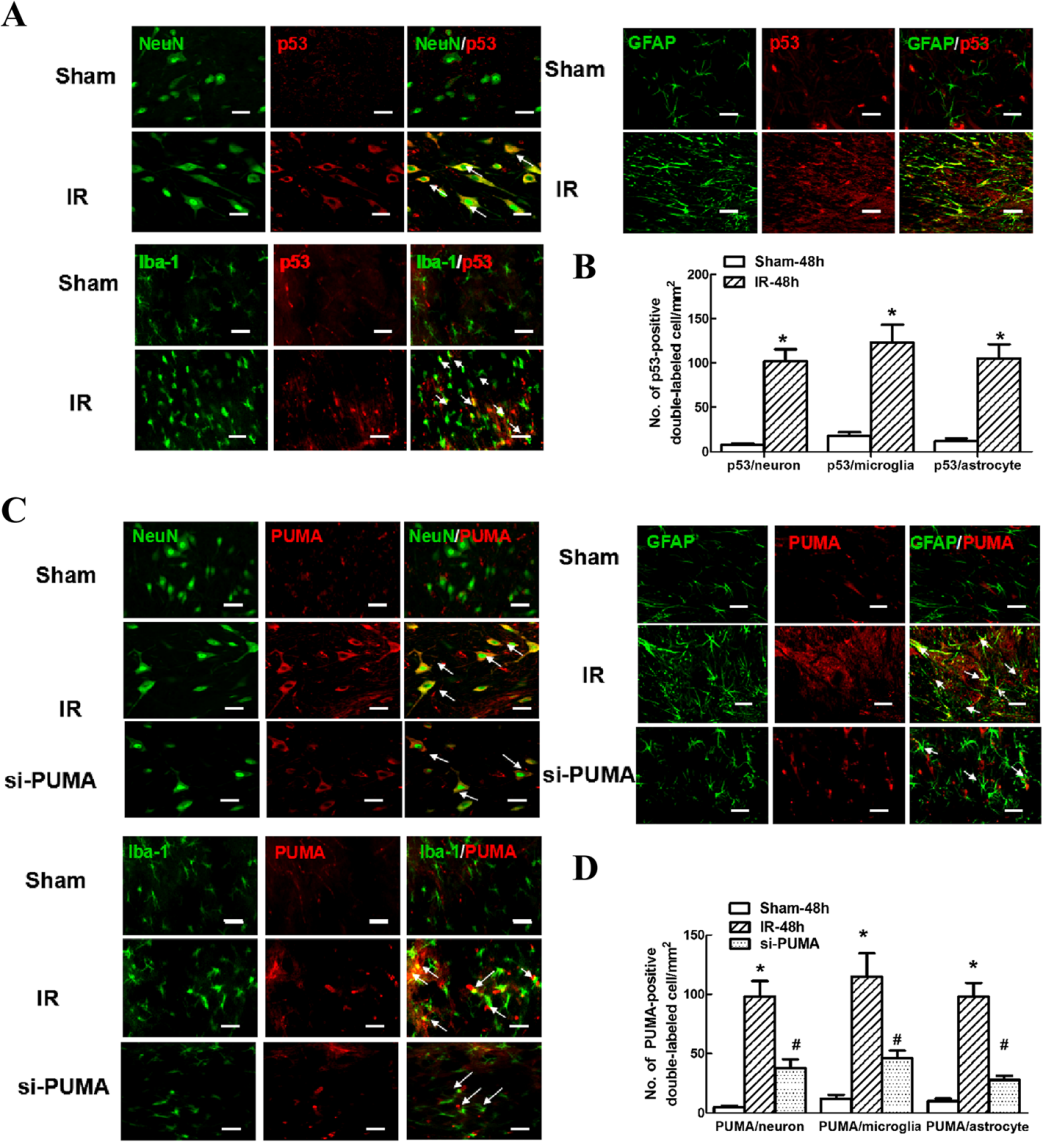
Double immunofluorescence staining of p53 and PUMA with spinal major cellular markers after IR. **a** Representative immunofluorescence analysis of the colocalization of neurons (NeuN; green), microglia (Iba1; green) and astrocytes (GFAP; green) with p53 (red) in the anterior horns of grey matter at 48 h after IR. The arrows indicate co-localization with yellow labelling. Scale bars = 50 μm. **b** Quantification of the p53-positive neurons, astrocytes and microglia was performed and presented as the average of three independent images. Data are expressed as the mean ± SEM. *P < 0.05, versus the sham group. **c** Representative immunofluorescence analysis of the colocalization of neurons (NeuN; green), microglia (Iba1; green) and astrocytes (GFAP; green) with PUMA (red) in the grey matter of the anterior horn at 48 h after IR with or without si-PUMA treatment. Scale bars = 50 μm. **d** Quantification of PUMA-positive neurons, astrocytes and microglia was performed and presented as the average of three independent images. Data are expressed as the mean ± SEM. *P < 0.05, versus the sham group. *P < 0.05, versus the IR group

To investigate the specific cellular changes in p53 and PUMA expression after IR, double immunofluorescence staining was performed at 48 h post-IR, at which point the maximal levels of p53 and PUMA were detected during reperfusion (Fig. 2). As shown in Fig. 3a, c, colocalization was displayed as a yellow fluorescent label under confocal microscopy. Compared with the sham group, significant increases in p53 and PUMA immunoreactivity were mainly distributed in cells positive for NeuN, GFAP and Iba-1 labelling, suggesting that p53 and PUMA were upregulated in both neurons and glial cells after IR. Similarly, quantitative analysis of the number of double-positive cells confirmed the above results (Fig. 3b, d, *P* < 0.05).

Additionally, compared with IR group, intrathecal pretreatment with si-PUMA showed markedly lower PUMA immunoreactivity in all cell types of the spinal cord (Fig. 3c and d, *P* < 0.05).

### Intrathecal injection of si-PUMA and PFT-α prevented p53 and PUMA upregulation after IR

**Fig. 4:**
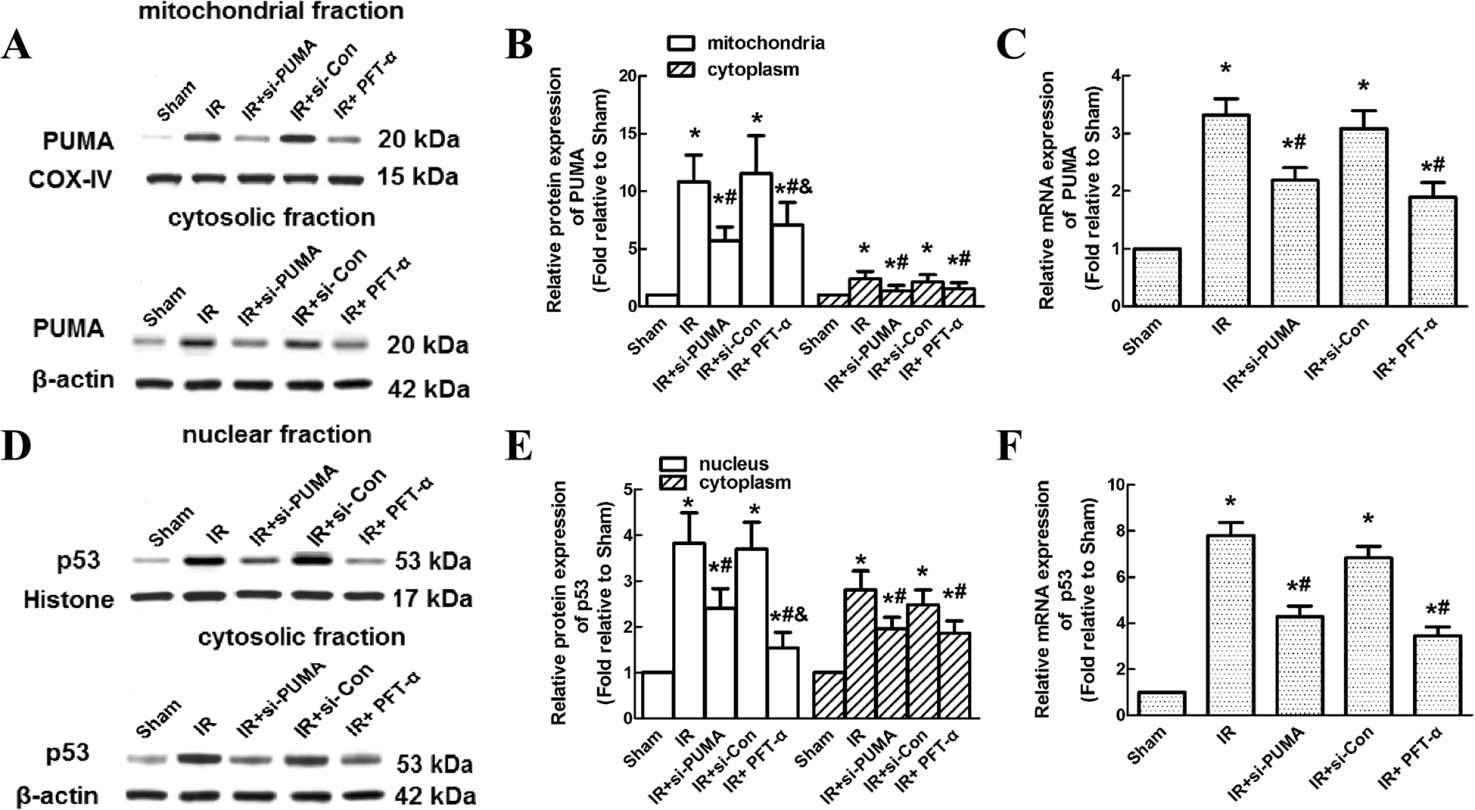
Effect of intrathecal si-PUMA and PFT-α injections on PUMA and p53 expression at 48 h after IR. **a** Representative western blots of mitochondrial and cytosolic PUMA protein levels at 48 h post-IR. COX-IV and β-actin were used as loading controls. **b** Quantification of the relative protein levels of PUMA in the mitochondria and cytoplasm. **c** Quantification of the relative mRNA expression of PUMA. **d** Representative western blots of nuclear and cytosolic p53 protein levels at 48 h post-IR. Histone and β-actin were used as loading controls. **e** Quantification of the relative protein levels of p53 in the nucleus and cytoplasm. **f** Quantification of the relative mRNA expression of p53. All data were obtained from three independent experiments and expressed as the mean ± SEM. *P < 0.05, versus the sham group. *P < 0.05, versus the IR group. *P < 0.05, versus the si-PUMA group

Because PUMA was shown to localize to mitochondria and induce cytochrome c release and subsequent caspase 3 activation [15], we detected PUMA levels in mitochondrial and cytosolic samples. As shown in representative western blots at 48 h post-IR, the intrathecal injection of si-PUMA and PFT-α significantly inhibited both the mitochondrial and cytosolic protein levels of PUMA, and these effects were not observed upon the injection of si-Con (Fig. 4a, *P* < 0.05). No significant differences were observed between the IR and si-Con groups (*P* > 0.05). Quantitative analysis of PUMA protein and mRNA expression, shown in Fig. 4b and c, confirmed the above results.

Given that p53 is the most important molecule upstream of PUMA and that the translocation of p53 from the cytoplasm to the nucleus signifies its activation, the protein levels of p53 in nuclear and cytoplasmic fractions were also determined. Compared with that in the IR group, the levels of p53 in both the nuclear and cytosolic samples were significantly decreased upon the intrathecal injection of si-PUMA and PFT-α (Fig. 4d and e, *P* < 0.05). Quantitative analysis of p53 protein and mRNA expression confirmed the above results (Fig. 4e and f, *P* < 0.05). No significant differences were observed between the IR and si-Con groups (*P* > 0.05). Interestingly, lower protein levels of p53 and PUMA were observed in rats injected with PFT-α compared to those in rats injected with si-PUMA (Fig. 4a, b, d and e, *P* < 0.05).

### Intrathecal injection of si-PUMA and PFT-α improved behavioural function by inhibiting neuroapoptosis and downregulating caspase 3

**Fig. 5:**
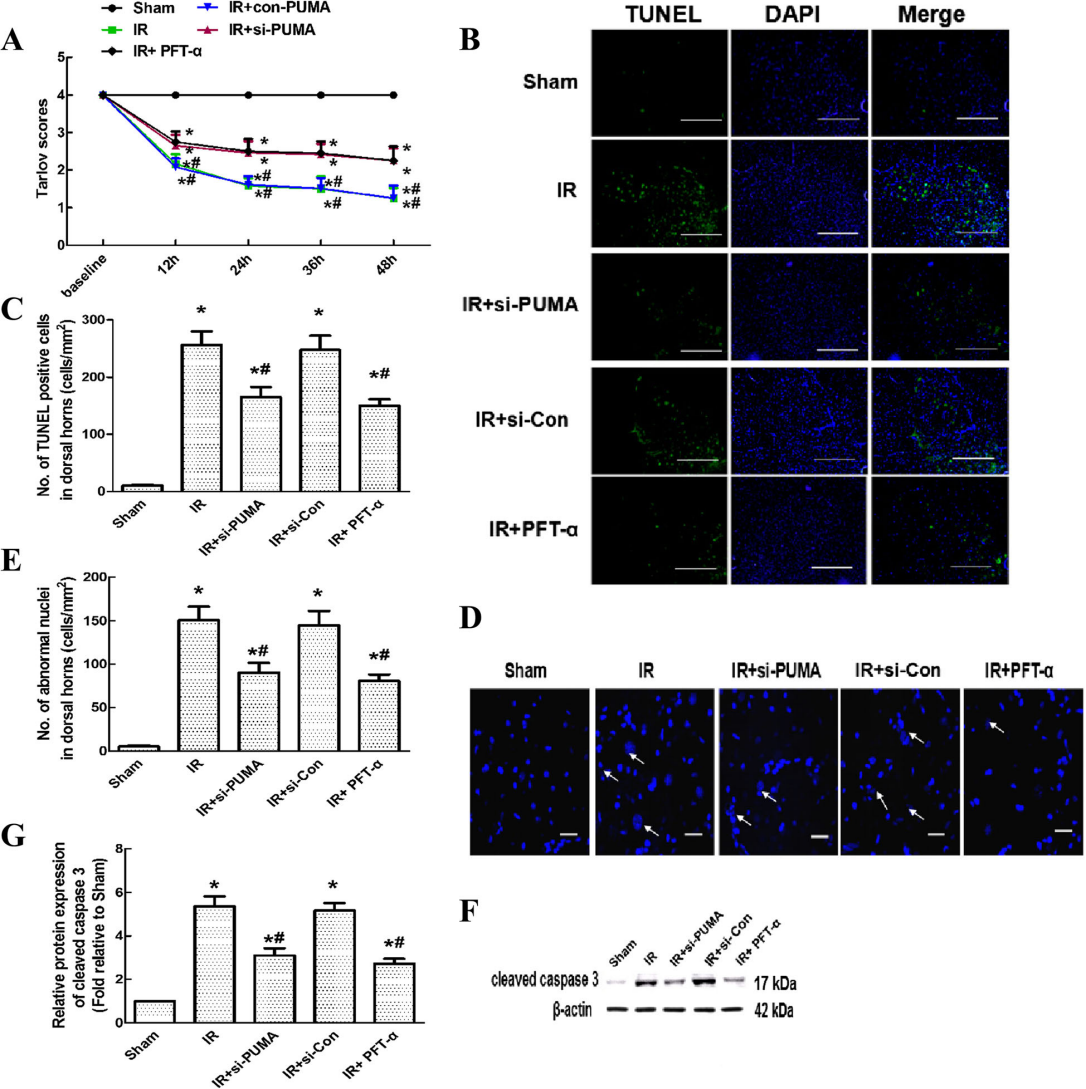
Effects of si-PUMA and PFT-α on behavioural function, neuroapoptosis and cleaved caspase 3 levels after IR. **a** Hind-limb motor function was assessed at 12-h intervals during the 48 h reperfusion period by Tarlov scores. **b** Representative micrographs of TUNEL (green) and DAPI (blue) staining in the anterior horns of grey matter at 48 h post-IR. Scale bars = 500 μm. **c** Quantification of the TUNEL-positive cells in the anterior horn as averaged from three independent experiments. **d** Representative micrographs of nuclear morphology as determined by staining with DAPI at 48 h post-IR. Scale bars = 50 μm. The arrows indicated the abnormal morphology of nuclei. **e** Quantification of morphological abnormalities in the nuclei of spinal anterior horns as averaged from three independent experiments. **f** Representative western blots of cleaved caspase 3 protein levels in the cytoplasm at 48 h post-IR. β-actin was used as the loading control. **g** Quantification of the relative protein level of cleaved caspase 3. Data are expressed as the mean ± SEM. *P < 0.05 versus the sham group. *P < 0.05 versus the IR group

Compared with those in sham-operated rats, all rats undergoing IR displayed hind-limb motor deficits, resulting in reduced Tarlov scores during the 48-h reperfusion period (Fig. 5a, *P* < 0.05). Compared with those for untreated rats in the IR group, rats injected with si-PUMA and PFT-α had significantly higher average Tarlov scores (*P* < 0.05), whereas the rats injected with si-Con had comparable scores (*P* > 0.05). No significant differences were observed between the IR and si-Con groups (*P* > 0.05).

Based on previous studies [3, 22], cellular apoptosis is a common cause of behavioural impairment. Thus, we further assessed whether the PUMA-p53 loop helps regulates apoptosis using the TUNEL assay and measurement of cleaved caspase 3 levels. As shown in representative TUNEL staining, the green fluorescent spots indicated cellular apoptosis (Fig. 5b). Quantitative analysis showed significantly more TUNEL-positive cells in the IR injury groups than in the sham group (Fig. 5c, *P* < 0.05). Compared with that in the IR group, increases in the number of TUNEL-positive cells in groups intrathecally injected with siRNA and PFT-α were significantly prevented (*P* < 0.05), whereas injection with si-Con did not induce these reductions (*P* > 0.05). These results were further confirmed by analysing the nucleic morphology by DAPI staining. As shown in Fig. 5d, normal nuclei were uniformly stained without condensation, and apoptotic nuclei displayed typical morphological features, such as abnormal nuclear size and nuclear fragmentation with some scattered apoptotic bodies. Similar to the TUNEL results, the number and morphology of nuclear abnormalities induced by IR were greatly ameliorated by the injection of siRNA and PFT-α (Fig. 5d and e, *P* < 0.05) but not by the injection of si-Con (*P* > 0.05).

Moreover, the levels of cleaved caspase 3, a well-known apoptotic marker, were detected by western blotting [22]. As shown in Fig. 5f and g, representative blots and quantitation showed that the patterns of cleaved caspase 3 protein levels among these groups were altered in a manner similar to those in the TUNEL assay (*P* < 0.05). No significant differences were observed between the IR and si-Con groups (*P* > 0.05).

### Intrathecal injection of si-PUMA and PFT-α inhibited neuroinflammation by inhibiting NF-κB-cytokine release after IR

**Fig. 6:**
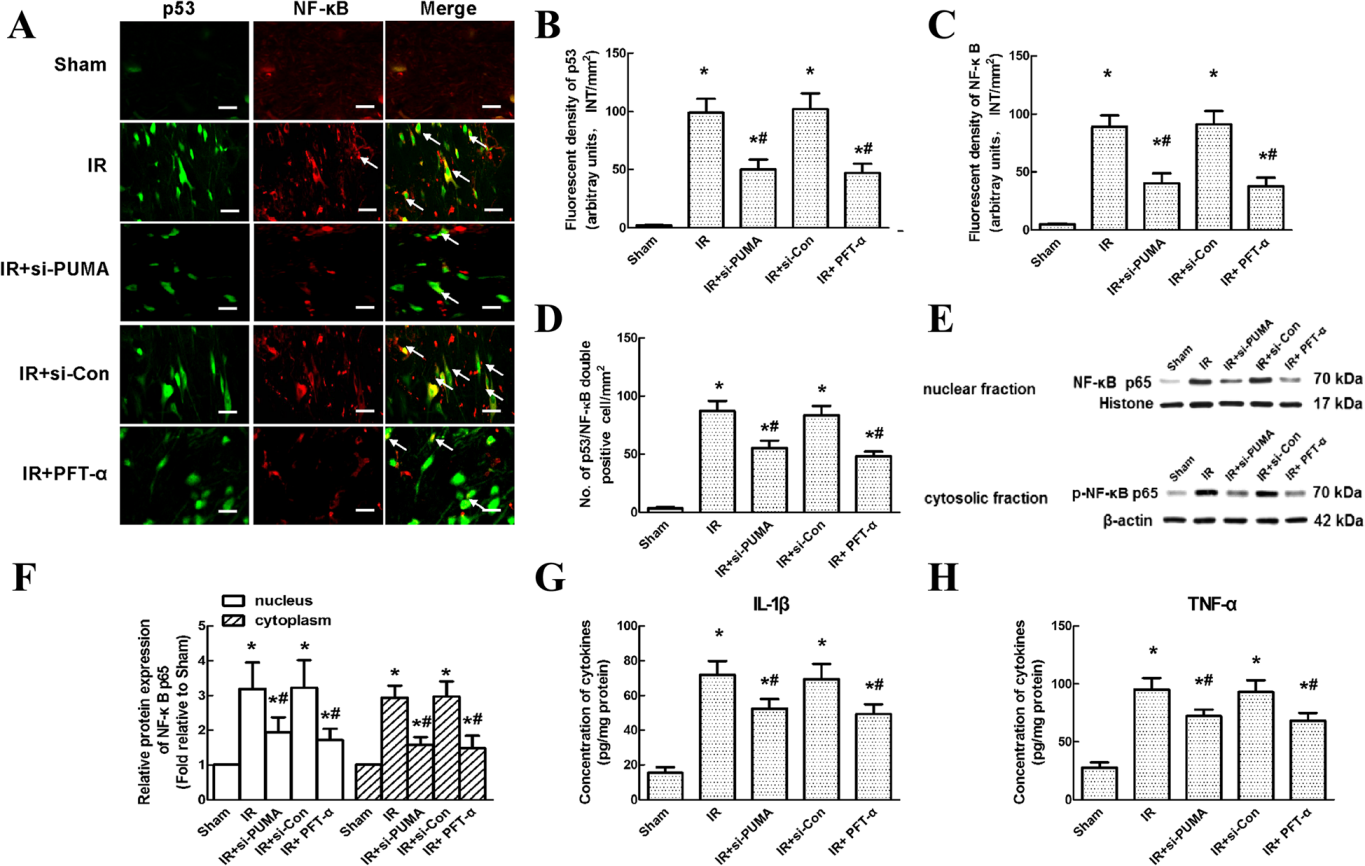
Effects of si-PUMA and PFT-α on NF-κB translocation and proinflammatory cytokine release after IR. **a** Representative immunofluorescence of the colocalization of p53 (green) and NF-κB (red) in the anteriorl horns of grey matter at 48 h after IR. The arrows indicate co-localization with yellow labelling. Scale bars = 50 μm. **b and c** Quantitation of p53 and NF-κB immunoreactivity in the anteriorl horns of grey matter at 48 h post-IR. **d** Quantification of p53/NF-κB double-positive cells is presented as the average of three independent images. **e** Representative western blots of the nuclear protein and cytosolic phosphorylation levels of NF-κB at 48 h post-IR. Histone and β-actin were used as loading controls. **f** Quantification of the relative protein levels of NF-κB in the nucleus and cytoplasm. **g and h** Quantification of spinal cord IL-1β and TNF-α concentrations at 48 h after IR. Data are expressed as the mean ± SEM. *P < 0.05, versus the sham group. *P < 0.05, versus the IR group

A previous chromatin immunoprecipitation assay showed that p53 and NF-κB p65 form a complex on the p53-responsive MDM2 promoter, and further activation of NF-κB is linked to p53 phosphorylation [23]. Thus, the role of the PUMA-p53 loop in regulating neuroinflammation after spinal cord IR was hypothesized to involve NF-κB activation. As shown by double immunofluorescence staining, the fluorescent p53 labels were consistently distributed with the NF-κB labels (Fig. 6a). Compared with those in the sham group, the immunoreactivities of p53 and NF-κB were much stronger in the IR group (Fig. 6b and c, *P* < 0.05). In addition, injection of si-PUMA and PFT-α significantly decreased both p53 and NF-κB immunoreactivity as well as the number of p53/NF-κB double-positive cells (Fig. 6b–d, *P* < 0.05), whereas no such changes were observed in the si-Con group (*P* > 0.05). Representative western blots further confirmed the above results. The nuclear localization and cytosolic phosphorylation of the p65 sub-unit have been used to indicate NF-κB activation [24]. Quantitative analysis showed that compared with those in the IR group, both the nuclear protein levels and cytosolic phosphorylation levels of NF-κB were significantly inhibited in the groups injected with si-PUMA and PFT-α (Fig. 6e and f, *P* < 0.05), whereas no significant differences were observed in the si-Con group (*P* > 0.05). Similarly, downstream products of the NF-κB pathway were assessed by measuring the IL-1β and TNF-α concentrations. Consistently, alterations in the IL-1β and TNF-α concentrations were similar to those in NF-κB protein levels (Fig. 6 g and h, *P* < 0.05).

### Intrathecal injection of si-PUMA and PFT-α preserved BSCB integrity after IR

**Fig. 7:**
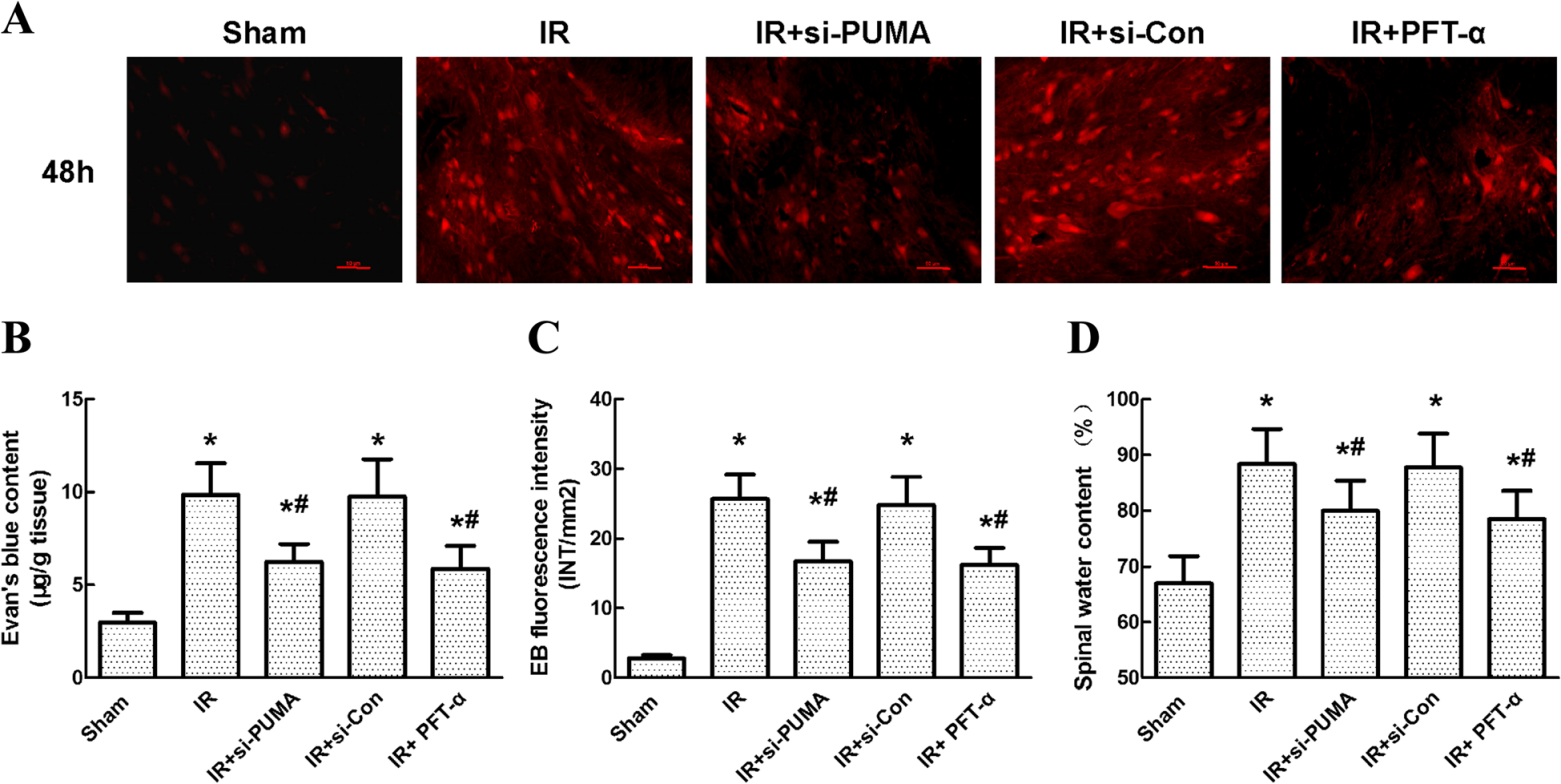
Effects of si-PUMA and PFT-α on blood-spinal cord barrier (BSCB) integrity after IR. **a** Representative images of IR-induced BSCB breakdown as measured by EB extravasation, which was visualized as red fluorescent spots under a microscope. **b** Quantification of EB content in the injured spinal cord presented as microgram per gram tissue. **c** Quantification of EB fluorescence intensity (INT/mm^2^). **d** Quantification of spinal water content (%). Data were obtained from three independent experiments and expressed as the mean ± SEM. *P < 0.05, versus the sham group. *P < 0.05, versus the IR group

EB extravasation was visualized as red spots under a fluorescence microscope. The representative images in Fig. 7a show that almost no red fluorescent spots were observed in the sham group. Compared with that in the sham group, a widespread distribution of red fluorescent spots that were much more intense was detected in the IR group. Injection of si-PUMA and PFT-α significantly decreased the number and intensity of the red spots. No differences were detected between the IR and si-Con groups (*P* > 0.05). Quantitation of the EB content and fluorescence intensity confirmed the above results (Fig. 7b and c, *P* < 0.05).

Meanwhile, IR-induced increases in water content are greatly attributed to BSCB breakdown [21]. The spinal cord water content was also changed in a manner similar to that of EB extravasation (Fig. 7d, *P* < 0.05).

## Discussion

The important and diverse roles of p53 and its driven targets under stimuli have been widely explored in previous studies [6–8]. Under pathological conditions, several p53-mediated pathways are quickly activated and further funnelled into the common node of the p53 network via MDM2 to balance the survival or death of cells [7, 25]. We demonstrated for the first time that PUMA, a target directly by p53 driven, also play crucial roles in spinal cord IR in addition to MDM2. Comprising the feedback loop, PUMA expression regulates the transactivation function of p53, which may further affect caspase 3-mediated apoptosis [15, 17, 26]. As expected, intrathecal treatment with si-PUMA and PFT-α exerted similar effects on preventing increased p53 and caspase 3 protein expressions. Additionally, the broader role of p53 in integrating with inflammatory pathways via NF-κB transcription has been reported [11]. Consistent with our study, comparable anti-inflammatory effects were also observed in rats injected with si-PUMA and PFT-α, as similar preservations of BSCB integrity and decreased protein levels of NF-κB and cytokines IL-1β and TNF-α were observed. Together, these results suggested that the p53-PUMA feedback loop functions as a common node to regulate cellular apoptosis and neuroinflammation during spinal cord IR.

Apoptosis is now known to be the most important factor related to neuronal loss during IR [22]. PUMA, a well-known p53-induced BH3-only protein, has been well demonstrated to regulate the apoptosis-promoting activity of tumour cells [27, 28]. PUMA is expressed at a very low level under normal conditions, and upregulating PUMA expression using the adenoviral gene delivery technique in ovarian cancer cells is thought to exert better apoptosis-inducing effects but lower proliferation-inhibiting effects than those in cells transfected with the p53 gene [27]. Simultaneously, PUMA is closely related to p53 activity, as they compose the feedback loop [16, 17]. PUMA expression is induced immediately after p53 translocation to the nucleus and consequently activates the execution of neuronal and myocardial apoptosis via the caspase cascade [15, 28, 29]. Silencing PUMA expression in cultured neurons prevented apoptosis to an extent similar to that in p53-deficient neurons [30]. A possible explanation for this phenomenon might be the decreased ability of PUMA to interact with p53, which, in turn, prevents p53 translocation and its transactivation ability to trigger cytochrome c release and activate caspase 3 [16, 17, 28]. In an in vitro study exploring the binding properties of p53 with PUMA and NOXA using a GST pull-down assay, the his-tagged p53 fusion protein was pulled down effectively with the BH3s of GST-PUMA and GST-NOXA, indicating that both the PUMA and NOXA BH3 regions interact with p53 in vitro [28]. Moreover, using the modelled complex structures, the predicted interaction sites further confirmed the structural interaction between p53 and PUMA [28]. Thus, asserting PUMA as a new treatment target in the regulation of cellular apoptosis during spinal cord IR is reasonable. Consistent with our study, PUMA and p53 protein are expressed at very low levels in normal spinal cord tissues. They are similarly rapidly increased with time over the same reperfusion period. To further clarify their interactions, we blocked PUMA and p53 functions by intrathecally injecting si-PUMA and PFT-α when their expression levels peaked. The decreased PUMA protein expression was changed consistently with the decreased p53 level. No significant differences were observed between treatment with si-PUMA and PFT-α (Fig. 4). In addition, some studies found that PUMA localized in mitochondria can decrease the ratio of the anti-apoptotic Bcl-2 protein to the pro-apoptotic Bax protein and then induce caspase activation [15, 30]. Thus, we also detected the protein levels of PUMA and p53 in subcellular fractions in this study. As shown by western blot analysis at 48 h post-IR, the protein levels of PUMA in the mitochondrial fraction were substantially increased at 48 h post-IR. In line with previous observations, the translocation of p53 to the nucleus indicated p53 transactivation [13, 15, 30]. As mutual factors in the feedback loop, the nuclear level of p53 was simultaneously significantly increased. Not surprisingly, downregulating PUMA via si-PUMA prevented increased mitochondrial PUMA and nuclear p53 levels to an extent similar to that achieved using the p53-specific inhibitor PFT-α. These results confirmed the feedback regulatory effects on PUMA and p53 during spinal cord IR and that PUMA directly regulates the p53-PUMA feedback loop.

p53 and its driven proteins are clearly activated in a tissue or cell type-specific manner, thus accounting for the variability in the diverse mechanisms regulated by p53 and the proteins it drives [25, 30]. In neurons, IR-induced damage can overwhelm repair systems and initiate caspase-mediated neuronal apoptosis [14, 22]. As shown herein, upregulated p53 and PUMA fluorescent labels colocalized with neuronal labels at 48 h post-IR (Fig. 3). Having many typical and common characteristics of currently known caspases, caspase 3 plays a crucial role in the execution phase of cellular apoptosis [22]. To further determine whether the p53-PUMA loop regulates neuronal apoptosis, TUNEL staining and cytosolic caspase 3 levels were examined simultaneously. A significant level of apoptosis was induced as the levels of PUMA and p53 increased, demonstrated as decreased Tarlov scores, but the number of TUNEL-positive cells and cleaved caspase 3 protein levels increased. Consistent with a previous in vivo study, transgenic mice carrying a PUMA mutation were protected against an apoptotic stimulus similarly to p53-deficient mice [31]. In the present study, intrathecal treatment with si-PUMA and PFT-α exerted similar effects on maintaining Tarlov scores and preventing increases in TUNEL-positive cells and cleaved caspase 3 levels in the spinal cord. In accordance with the abovementioned biochemical changes, our morphological evaluation by DAPI staining further confirmed that si-PUMA and PFT-α treatment similarly reduced the number of nuclei with typically apoptotic morphology, underscoring the decreased ability of apoptosis due to inhibition of the PUMA-p53 feedback loop.

Neuroinflammation is another major crucial factor contributing to the pathophysiological mechanisms of IR [18, 21, 22]. Emerging evidence suggests broader roles of p53 in interacting with the inflammatory pathway by modulating glial function and promoting NF-κB transcription [11, 32, 33]. For example, microglial secretion of the inflammatory cytokines IL-1β and TNF-α was demonstrated to be p53-dependent in neurodegenerative diseases, whereas the inhibition of p53 by PFT-α reversed the above proinflammatory phenotypic change in microglial cells [32]. Consistent with this observation, our double immunofluorescence staining confirmed that increased p53 and PUMA fluorescent labels were also widely distributed in activated astrocytes (displayed as hypertrophic body size) and microglial cells (displayed as amoeboid morphology). Moreover, the increased fluorescent p53 labels coincidentally localized with the NF-κB labels, indicating their potential functional crosstalk in IR-induced inflammatory responses (Fig. 6). In the NF-κB signalling cascade, phosphorylation is the essential step for the translocation of NF-κB from the cytoplasm to the nucleus, which then activates downstream transcription [24]. Thus, the nuclear localization and/or the use of phospho-specific antibodies to detect phosphorylation levels are two common methods for detecting NF-κB activation. Consistent with our previous studies in vivo, the increased nuclear translocation of NF-κB and glial production of cytokines are the quintessential markers of NF-κB-mediated neuroinflammation in injured spinal cords [34]. Recently, one study suggested that the different phosphorylation sites of the p65 sub-unit result in different levels of NF-kB transcriptional activity. Neither phosphorylation in cytoplasm nor translocation to nucleus alone is not sufficient to indicate NF-kB transcription. It was important to assess both, especially in the absence of an NF-κB transcription-dependent functional read out [24]. Thus, we assessed both indexes in this study. As shown in Fig. 6, the number of p53/NF-κB double-labelled cells, the nuclear protein and cytosolic phosphorylation levels of NF-κB and the accompanying levels of IL-1β and TNF-α were all greatly inhibited by treatment with si-PUMA and PFT-α. Additionally, some published articles have emphasized that BSCB integrity is another useful factor for evaluating neuroinflammation after IR [18, 21]. Increased BSCB leakage can easily be recognized by substantial increases in spinal water content and EB extravasation [21]. As expected, substantially improved BSCB integrity was achieved by controlling the cytokine content by treatment with si-PUMA and PFT-α. These findings provided clear evidence of the effects of the p53-PUMA feedback loop on neuroinflammation after IR.

Notably, contradictory p53 expression has been observed in astrocytes [14, 35]. For example, p53 was not obviously detectable in activated astrocytes at 24 h or 72 h after ischaemia but was clearly induced at 7 days post-injury [14]. These discrepancies were most likely attributed to the use of different disease models and the time points utilized to study specific glial cell activation. In addition, sequential activation and crosstalk between microglia and astrocytes are common in vivo, but they do not always occur in the same order given the diverse mechanisms of diseases [36]. Additionally, neuron-glial signalling exists not only at synaptic and axonal contacts but also via direct interaction with neuronal cell bodies [37]. In this context, the complexity of the neuron-glia architecture in the mammalian CNS greatly hindered the detailed determination of the neuron-glia pathophysiological functions and interactions in vivo*.* Therefore, in this study, we only confirmed the cellular location of p53 and PUMA in spinal cells and explored the net effects of si-PUMA treatment in rats by detecting the BSCB integrity and hind-limb motor function, regardless of how si-PUMA was regulated among spinal cells. Further in vitro studies are needed to investigate the mechanism underlying the p53-PUMA feedback loop in regulating other mediators to better elucidate its common node in multiple IR mechanisms.

## Conclusions

In conclusion, this study explored the roles of p53 and its driven target PUMA in spinal cord IR. Inhibition of aberrant p53-PUMA feedback loop activation by the intrathecal injection of si-PUMA and PFT-α prevented IR-induced apoptosis, inflammatory responses and BSCB breakdown by inhibiting the activation of caspase 3-mediated apoptosis and NF-κB-mediated cytokine release. These results suggest potential new strategies for IR treatment.

## Abbreviations

Bcl-2: B-cell lymphoma-2
Bid: BH3-interacting domain death agonist
BSCB: Blood-spinal cord barrier
DAPI: 4′,6-diamidino-2-phenylindole
DMSO: Dimethyl sulfoxide
IL: Interleukin
IR: Ischemia reperfusion
MDM2: Murine double minute 2 gene
NF: Nuclear factor
PBS: Phosphate-buffered saline
PUMA: p53 upregulated modulator of apoptosis
TNF: Tumor necrosis factor
TUNEL: Terminal deoxynucleotidyl-transferase mediated dUTP nick-end labelling
