# Correction to: Inhibiting aberrant p53-PUMA feedback loop activation attenuates ischaemia reperfusion-induced neuroapoptosis and neuroinflammation in rats by downregulating caspase 3 and the NF-κB cytokine pathway

**DOI:** 10.1186/s12974-021-02344-3

**Published:** 2021-12-28

**Authors:** Xiao-Qian Li, Qian Yu, Feng-Shou Chen, Wen-Fei Tan, Zai-Li Zhang, Hong Ma

**Affiliations:** 1 Department of Anesthesiology, First Affiliated Hospital, China Medical University, Shenyang, 110001 Liaoning China; 2 Department of Thoracic Surgery, Fourth Affiliated Hospital, China Medical University, Shenyang, 110032 Liaoning China

## Correction to: Journal of Neuroinflammation (2018) 15:250 10.1186/s12974-018-1271-9

The original version of the article [1] unfortunately contained a mistake. The error occurred in representative images of double immunofluorescence of Figure 3.

The other data and the conclusion in the publication are real and reliable.

It has been corrected in this correction.

The correct version of Fig. 3 is given in this erratum.

**Fig. 3:**
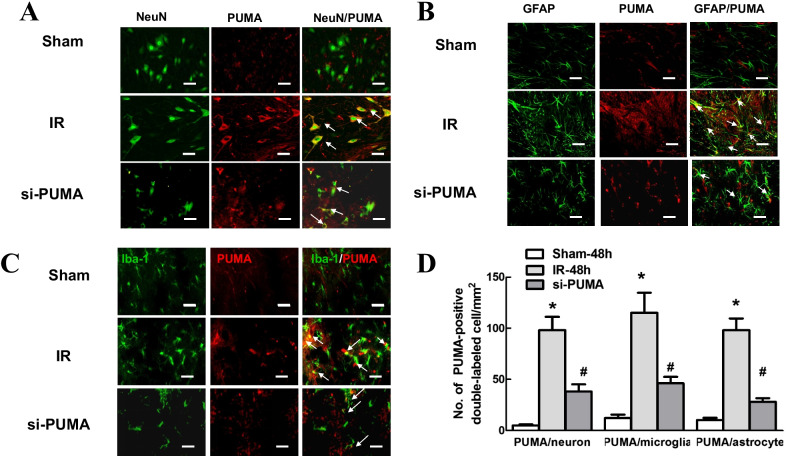
Double immunofluorescence staining of p53 and PUMA with spinal major cellular markers after IR. **a** Representative immunofluorescence analysis of the colocalization of neurons (NeuN; green), microglia (Iba1; green) and astrocytes (GFAP; green) with p53 (red) in the anterior horns of grey matter at 48 h after IR. The arrows indicate co-localization with yellow labelling. Scale bars = 50 μm. **b** Quantification of thep53-positive neurons, astrocytes and microglia was performed and presented as the average of three independent images. Data are expressed as the mean ± SEM. *P < 0.05, versus the sham group. **c** Representative immunofluorescence analysis of the colocalization of neurons (NeuN; green), microglia (Iba1; green) and astrocytes (GFAP; green) with PUMA (red) in the grey matter of the anterior horn at 48 h after IR with or without si-PUMA treatment. Scale bars = 50 μm. **d** Quantification of PUMA-positive neurons, astrocytes and microglia was performed and presented as the average of three independent images. Data are expressed as the mean ± SEM. *P < 0.05, versus the sham group. *P < 0.05, versus the IR group
